# Prevalence of colonization by multiresistant bacteria on admission to the ICU in the French military hospital in Kabul, Afghanistan

**DOI:** 10.1186/cc12016

**Published:** 2013-03-19

**Authors:** JV Schaal, P Pasquier, H Delacour, A Salvadori, A Jarrassier, JR Renner, SM Mérat

**Affiliations:** 1 Military Teaching Hospital Bégin, Saint-Mandé, France

## Introduction

The French military hospital at the Kaboul International Airport (KaIA) base provides surgical care for International Force and Afghan National Army soldiers, and also local patients. The development of multiresistant bacteria (MRB) nosocomial infections has raised a major problem complicating the care of combat casualties [1]. The aim of this study is to assess the prevalence of MRB carriage on admission to the ICU in this combat support hospital.

## Methods

We used a prospective observation study on patients admitted to the French military ICU in KaIA over 3 months (July to September 2012). All hospitalized patients were assessed for the presence of colonization with MRB: nasal and rectal swabs were performed to identify, respectively, methicillin-resistant *Staphylococcus aureus *(MRSA) and extended-spectrum ß-lactamases bacteria (ESBLB). The following data were recorded for each patient on admission: demographic characteristics, bacteriological results, length of stay, type of previous hospitalization.

## Results

Sixty-three patients were admitted. The mean length of stay (MLS) was 3 ± 3 days, and the mean age was 25 ± 14 (13 patients <15 years). Patients were hospitalized for combat-related trauma (74%), noncombat-related trauma, medical pathologies (10%), and postoperative care (8%). They were Afghans (92%) or westerners (8%). Swabs were not realized for eight patients. Forty-three percent revealed an ESBLB colonization: *Escherichia coli *(22 patients), *Klebsiella pneumoniae *(one patient), *Acinetobacter baumanii *(one patient). No patients were colonized with MRSA. Ten patients (16%) were directly admitted to the ICU, 12 (19%) had been hospitalized before admission, 39 (62%) were transferred after resuscitative and stabilization care in a level 2 unit. For the two last categories, the MLS (for previous hospitalization) was respectively 14 ± 28 days and 8 ± 6 hours. Among patients transferred after care in a level 2 unit, MLS was no different between colonized and noncolonized patients: 8 ± 7 versus 9 ± 6 hours (*P *= 0.5, Mann-Whitney test).

## Conclusion

In this study, prevalence of colonization with ESBLB at admission is very high, suggesting a high prevalence of MDR colonization in the local population in Afghanistan. It remains important to intensify the prevention policy against MRB cross-transmission in the deployed ICU.
